# Physical/mechanical and antibacterial properties of composite resin modified with selenium nanoparticles

**DOI:** 10.1186/s12903-024-04965-5

**Published:** 2024-10-19

**Authors:** Sara Khaled ElSheikh, El-Sayed Gad Eid, A. M. Abdelghany, Dina Abdelaziz

**Affiliations:** 1 Department of Dental Biomaterials, Faculty of Oral and Dental Medicine, Delta University for Science and Technology, Dakahlia Governorate, Egypt; 2 Department of Dental Biomaterials, Faculty of Dentistry, Mansoura University, El Gomhouria St, Mansoura, Dakahlia 35516 Egypt; 3 Spectroscopy Department, Physics Research Institute, National Research Centre, 33 Elbehouth st., Dokki, Giza 12311 Egypt

**Keywords:** Selenium nanoparticles, Composite resin, Antibacterial, Biofilms, Mechanical, Surface roughness, Color difference

## Abstract

**Background:**

Accumulation of biofilm over composite resin restorations is one of the principal causes of recurrent caries. Therefore, this study aimed to develop antibacterial composite resins by crystalline selenium nanoparticles (SeNPs), assessing the antibacterial, mechanical, and physical properties of the composite resin after SeNPs incorporation.

**Methods:**

SeNPs were synthesized via a green method. The nanoparticles were characterized by UV-Vis spectroscopy, fourier transform infrared (FT-IR) spectroscopy and transmission electron microscopy (TEM). The nano-filled composite (Filtek™ Z350XT ) was considered as a control group (G0). Two concentrations of SeNPs (0.005 wt% and 0.01 wt%.) were added to the tested resin composite (G1& G2), respectively. The physical/mechanical and antibacterial properties of the composite specimens (*n* = 10/group) were characterized. A one-way ANOVA was conducted to analyze these data followed by Bonferroni post hoc test for pairwise comparison.

**Results:**

Modified composites with SeNPs showed antibacterial activity against *E. coli* and *S. mutans.* Mechanical properties including diametral tensile strength, compressive strength, or surface roughness were not affected by nano-incorporation compared to control. Furthermore, the degree of conversion showed no statistical difference. However, SeNPs incorporation into resin composite produces color change that can be visually perceived.

**Conclusions:**

The green synthesized SeNPs significantly improved the antimicrobial properties of the dental composite without compromising mechanical performance. However, it shows color change after SeNPs incorporation.

## Background

Tooth-colored materials have been progressively used in dentistry as aesthetics becomes the prime requirement. Therefore, composite resins are widely used for dental restorations due to their esthetics, mechanical strength, and direct filling capabilities [1]. Dental composite resin materials have two main constituents: Resin matrix and ceramic fillers. The resin matrix is composed of monomers, diluents, photo initiators, accelerators, and coupling agents. Recently, nano particles and nano fibers are typically utilized as fillers due to their excellent aesthetic, bioactivity, and biocompatibility properties [2]. Nevertheless, more biofilm are accumulated on composite resin restorative materials than other restorative materials with subsequent secondary caries, restoration failure and finally, replacement of restorations [3, 4]. Nanotechnology initiates great advances in biological and biomedical science. The use of nanoparticles to improve properties of dental restorative materials has been increased. Particularly, development of restorative materials with anticaries properties becomes the scope of researchers [5, 6]. Hence, the incorporation of nanoparticles into the resin-based restorative materials has been increasingly used to achieve antibacterial activity [7, 8]. 

Nanoparticles can be fabricated through several approaches, including physical, chemical, and biological methods. Top-down methods include breaking down bulk materials, while bottom-up approaches build nanoparticles from the atomic or molecular scale [9, 10]. Though widely used, conventional physical and chemical routes have faced limitations regarding cost, time, and use of hazardous chemicals. However, green synthesis techniques provide safer, eco-friendly, and economical alternatives by utilizing plant extracts or biological systems. They overcome key issues faced in other nanoparticle production methods, which is especially vital for biomedical applications. Hence, green approaches enable straightforward and sustainable nanoparticle synthesis without the constraints present in certain top-down and earlier bottom-up techniques. In recent years, numerous studies have focused on green nanoparticle production methods as favorable substitutes for certain physical and chemical manufacturing processes [11, 12]. 

Selenium (Se) is an essential trace element directly involved in several biological activities, involving cellular division and differentiation, antioxidant defense mechanism, and preservation of appropriate immune response [13]. The human body has over 25 selenoproteins and adequate levels of Se are necessary for the adequate induction of the immune response [14]. Hence, selenium has been broadly employed in the medical field as an antioxidant, anti-inflammatory, anticancer, and antibacterial element [15]. Also, antibacterial effect of SeNPs has been demonstrated against *S. mutans* [16]. Using a green synthetic technique, spherical selenium (Se) nanostructures were generated using citric acid or plant extracts and showed significantly greater antibacterial activity than those manufactured chemically [17]. Moreover, the circumstances of the chosen manufacturing pathway may affect the physicochemical properties of SeNPs [18]. SeNPs developed using a natural source are substantially less hazardous than chemically stabilized nanoparticles. Until now, no study has employed selenium nanoparticles as an antibacterial filler for composite resin. However, development of dental composite with antimicrobial activity through incorporation of nanoparticles could negatively influence the physical and mechanical properties [19]. A further drawback might be the reduced color stability gained in materials incorporating nanoparticles, rendering these materials clinically undesirable especially in case of aesthetic materials [20]. 

Consequently, the presented work demonstrates modification of a commercial composite resin with green synthesized SeNPs to impart antibacterial properties against *E. coli* and *S. mutans* without surrendering the mechanical and physical properties. The study’s null hypothesis is that the incorporation of SeNPs into a nanofilled composite resin: (1) does not impart antibacterial activity to composite resin, (2) does not change diametral tensile strength, compressive strength, surface roughness, degree of conversion, nor color.

## Materials and methods

### Materials

In this study, polyvinyl alcohol (PVA) was purchased from Merck, Germany. Ascorbic acid and sodium selenite were obtained from Sigma Aldrich, USA. Filtek™ Z350XT (3M USA) was used as the nanofilled composite resin.

### Sample size calculation

The sample size was assessed with power and sample size calculation software (PS, version 3.1.6) [20]. The sample size was established with at least 9 per group. The estimated difference was 0.8 when probability (power) was 0.8. Type I error probability accompanying this test was 0.05. To account for a potential 15% failure, the whole sample size is raised to 10 per group.

### Green synthesis and characterization of SeNPs

Selenium nanoparticles (SeNPs) were synthesized using a reduction reaction between sodium selenite and ascorbic acid, with a stabilizing agent of polyvinyl alcohol (PVA). Under magnetic stirring conditions for 5 min, 50 mM sodium selenite and 0.1 wt% PVA as stabilizing agents were combined to form stabilized SeNPs. The solutions were then incrementally supplemented with 1wt% ascorbic acid in deionized water. For full reduction, the reaction mixtures were stirred for 30 min. As the reducing agent was introduced, the colorless solutions gradually turned orange, indicating selenium nanoparticles were being formed [21]. 

UV-Vis absorption spectroscopy (Bio Aquarius CE 7250, UK) was used to characterize the produced NPs, followed by transmission electron microscopy (TEM) to examine appearance and size. Micrographs were taken with a Zeiss-EM10C-100KV electron microscope. Image J analysis software program was used to determine the particle size of individual particles in the TEM image. ThermoFisher-USA’s Nicolet IS10 instrument was used to perform Fourier transform infrared (FT-IR) spectroscopy on selenium nanoparticles in the spectral range 4000 –400 cm^− 1^ to identify and analyze the presence of specific chemical bonds (functional groups). X-ray diffraction (XRD) patterns of the specimens were determined by DIANO company USA with CuK radiation at the Bragg angle 2ϴ = 5^o^ to 80^o^.

### Composite resin used in this study

The nanofilled composite resin Filtek ^TM^ Z350XT (3 M USA) at color A2B (Body) was used as a control group and was compared by experimental groups modified by SeNPs. The composition of this composite resin is displayed in Table 1. The control group (G0) comprised seventy samples prepared from resin composite without additives. Additionally, 140 samples were prepared, divided into 2 equal groups (G1& G2), with 70 specimens each, based on nanoparticle concentration (0.005 wt% and 0.01 wt%.), respectively. The use of such low concentrations of selenium nanoparticles in dental resin composites represents a carefully balanced approach to introduce antibacterial properties while maintaining physicomechanical or aesthetic properties of the restorative material. Each group is subjected to characterization regarding its antibacterial activity, diametral and compressive strengths, surface roughness, degree of conversion, and color stability.

### Composite resin specimens’ preparation

The SeNPs were incorporated into the composite resin Filtek ^TM^ Z350XT (3 M USA) following a standardized protocol based on the inclusion of weight% of particles [22]. SeNPs concentrations (0.005 and 0.01 wt%) were prepared to be added to the tested resin composite. The required mass of nanoparticles was weighed for each gram of resin to achieve the desired concentration of nanoparticles in relation to composite resin. Then, SeNPs were blended into the composite resin for 1 min, using a metal spatula and a glass plate. The modified resin was placed inside a dark box to avoid accidental polymerization before placement in the mold. During preparation of composite resin specimens. First, a transparent mylar strip was applied against a microscopic glass slide, then the mold was fixed on it. After overfilling the mold, another mylar strip and a glass slide were pressed over the mold. The slide was pressed lightly and constantly to allow excess material to be extruded, resulting in a smooth surface. After removing the glass slide, the samples were cured for 30 s by the light-guide tip of the LED light cure with wavelength 400–500 nm, then the bottom slap was removed, and the curing was repeated from the other side. The light intensity of the lead light cure was regularly checked by radiometer (SDI LED Radiometer). The prepared samples were kept in distilled water for 24 h at 37 °C to ensure complete polymerization. To approve the homogenous distribution of nanoparticles within the composite resin, scanning electron microscopy (SEM, JEOL-6510LV) with an energy-dispersive X-ray mapping analysis (EDX, Oxford Xmax 20, England) was conducted on the fractured surface of one sample of each modified composite groups (G1 and G2).

### Evaluation of antibacterial properties

#### Microbial strain and growth media

The antibacterial activity was measured against *Streptococcus mutans* (NCTC No. 10449) and *E.coli* (EMCC No. 1815). Bacterial strains were transferred from stock culture to Brain Heart Infusion (BHI) broth and incubated at 37° C for 24 h. After incubation, the bacterial growth was measured by the presence of turbidity in the broth. Turbidity was evaluated visually by comparing the inoculum tube and the standards against a contrasting black card. The antibacterial test was then done using Agar disc diffusion test.

#### Agar disc-diffusion

Thirty disc-shaped samples, with 10 samples in each group, were made in a plastic split mold (4 mm diameter and 2 mm thickness). Specimens of each group were sterilized under ultraviolet light for 2 h before the antibacterial test. 1–2 × 10^8^ CFU/ml of bacterial suspension were placed on 150 mm of each solid agar plate. The composite resin discs were placed on the solid agar surface. All plates were maintained at room temperature for 2 h to permit diffusion of the test materials and then incubated at 37 ◦C for 24 h in an incubator. The diameter of the halo around the specimens (inhibition zone) was measured in each plate by the same operator to confirm the standardization and accuracy by millimeter ruler [19]. 

### Diametral tensile and compressive strength tests

A total number of 60 disc-shaped specimens (10 specimens for each group) were prepared in a plastic split mold (6 mm diameter and 3 mm thickness), (4 mm diameter and 6 mm thickness) for diametral tensile and compressive strength respectively. Diametral tensile strength was examined, and data were collected using computer software called Bluehill Lite from Instron^®^, and each sample was mounted separately on computer-controlled materials testing equipment (Model 3345; Instron Industrial Products, Norwood, MA, USA) equipped with a loadcell of 5 kN, operating at a cross-speed of 0.5 mm/min. The samples were statically loaded diametrically until fracture [19]. The maximum failure load was recorded in N. The diametral tensile strength was measured using this equation:

$$\delta \, = \,2{\rm{P}}\,/\,\pi \,{\rm{DT}}$$

Where; δ = Diametral compressive strength (MPa), P = load at failure (N), π = 3.14, D = disc diameter(mm), T = disc thickness(mm).

For the compressive strength test, the tested specimens were positioned with their flat ends between the plates of the Instron, and the compressive load was applied parallel to the long axis of the specimens. The compressive strength was calculated using this equation:

Compressive strength (CS) = 4P/πd2. Where P is the load (N) at the fracture point and d is the diameter (mm) of the sample [23]. 

### Surface roughness

Thirty disc-shaped samples, with ten samples in each group, were prepared in a plastic split mold (8 mm diameter and 2 mm thickness). An optical non-contact technique was employed to quantitatively examine surface topography. Samples were captured via a USB Digital microscope attached to a built-in camera (Scope Capture Digital Microscope, Guangdong, China) connected to IBM compatible computer at a magnification of 120X. The images were taken with a resolution of 1280 × 1024 pixels per image and after that cropped to 350 × 400 pixels with Microsoft Office picture manager to confirm standardization of measurement area. Subsequently, a 3D image of the specimens’ surface was generated after analyzing via WSxM software. Three 3D images were taken for each sample covering an area of 10 μm ×10 μm. WSxM software was operated to analyze average surface roughness (Ra) in mm [24]. 

### Evaluation of the degree of conversion (DC)

Thirty disc-shaped cured samples were prepared, with ten samples in each group, using a plastic split mold with a diameter of 5 mm and a thickness of 2 mm. After polymerization and 24 h of storage in distilled water, each sample was manually ground into fine powder with a mortar and pestle. Fifty micrograms of the ground powder were blended with potassium bromide (KBr) in an amount that is as ten times as the specimen powder. Then, powders were condensed into discs. These condensed disks were characterized by FTIR spectroscopy. Uncured thirty samples, with ten samples in each group, were also mixed with potassium bromide powder and pressed to obtain the pellets. The ratio of absorbance intensities of the aliphatic C = C double bond at 1638 cm^− 1^, and aromatic C….C double at 1608 cm^− 1^, is used to calculate the percentage of DC by using the following equation [25]:

$$DC\, = \,(1\, - \,R\,(cured)\,/\,R\,(uncured)\, \times \,100$$

### Evaluation of color difference

Thirty disc-shaped specimens (10 specimens for each group) were prepared in a plastic split mold (4 mm diameter and 2 mm thickness). A Spectrophotometer (X-Rite, model RM200QC, Neu34 Isenburg, Germany) was used to gauge each specimen’s color. The samples were positioned within the instrument against a white background at 4 mm from the aperture. Measurements were taken according to The International Commission on Illumination’s (CIE) standards illuminant D65 using the CIE L*a*b* color space [20]. The color difference (ΔE) of the specimens were calculated between the coordinates obtained from the samples without SeNPs (G0) and after SeNPs incorporation (G1 and G2) using the following equation [26]:

$$\Delta {\rm{E}}{\mkern 1mu} = {\mkern 1mu} {\left[ {{{\left( {\Delta {\rm{L}}*} \right)}^2}{\mkern 1mu} + {\mkern 1mu} {{\left( {\Delta {\rm{a}}*} \right)}^2}{\mkern 1mu} + {\mkern 1mu} {{\left( {\Delta {\rm{b}}*} \right)}^2}} \right]^{{\raise0.7ex\hbox{$1$} \!\mathord{\left/{\vphantom {1 2}}\right.\kern-\nulldelimiterspace}\!\lower0.7ex\hbox{$2$}}}}$$

Where: L* = lightness (0-100), a* = (color variation on the red/green axis) and b* = (color variation on yellow/blue axis).

### Statistical analysis

The normal distribution of data was evaluated by investigating the data distribution and employing Kolmogorov-Smirnov and Shapiro-Wilk tests. Data management and statistical analysis were performed using the Statistical Package for Social Sciences (SPSS) version 18. A one-way ANOVA was conducted to analyze the antibacterial activity, diametral tensile strength, compressive strength, surface roughness, degree of conversion, and color change regarding the three tested groups. Bonferroni post hoc test was used for pairwise comparison. A significant level of 5% was utilized for all statistical analyses.

**Table 1:** Composite resin used in the study

Material	composition	Manufacturer	Batch No.
Filtek™ Z350xt composite	Bis-GMA, UDMA, TEGDMA, Bis-EMA, Zirconia (4–11 nm ), nanosilica (20 nm), aggregated silica/zirconia cluster filler 78.5wt%	3 M Filtek, USA	775,639

BIS-GMA, Bisphenol A glycidyl methacrylate; UDMA, Urethane dimethacrylate; TEGDMA, Triethylene glycoldimethacrylate; BIS‑EMA, Bisphenol A ethoxylated dimethacrylate

## Results

### Characterization of prepared SeNPs

**Fig. 1:**
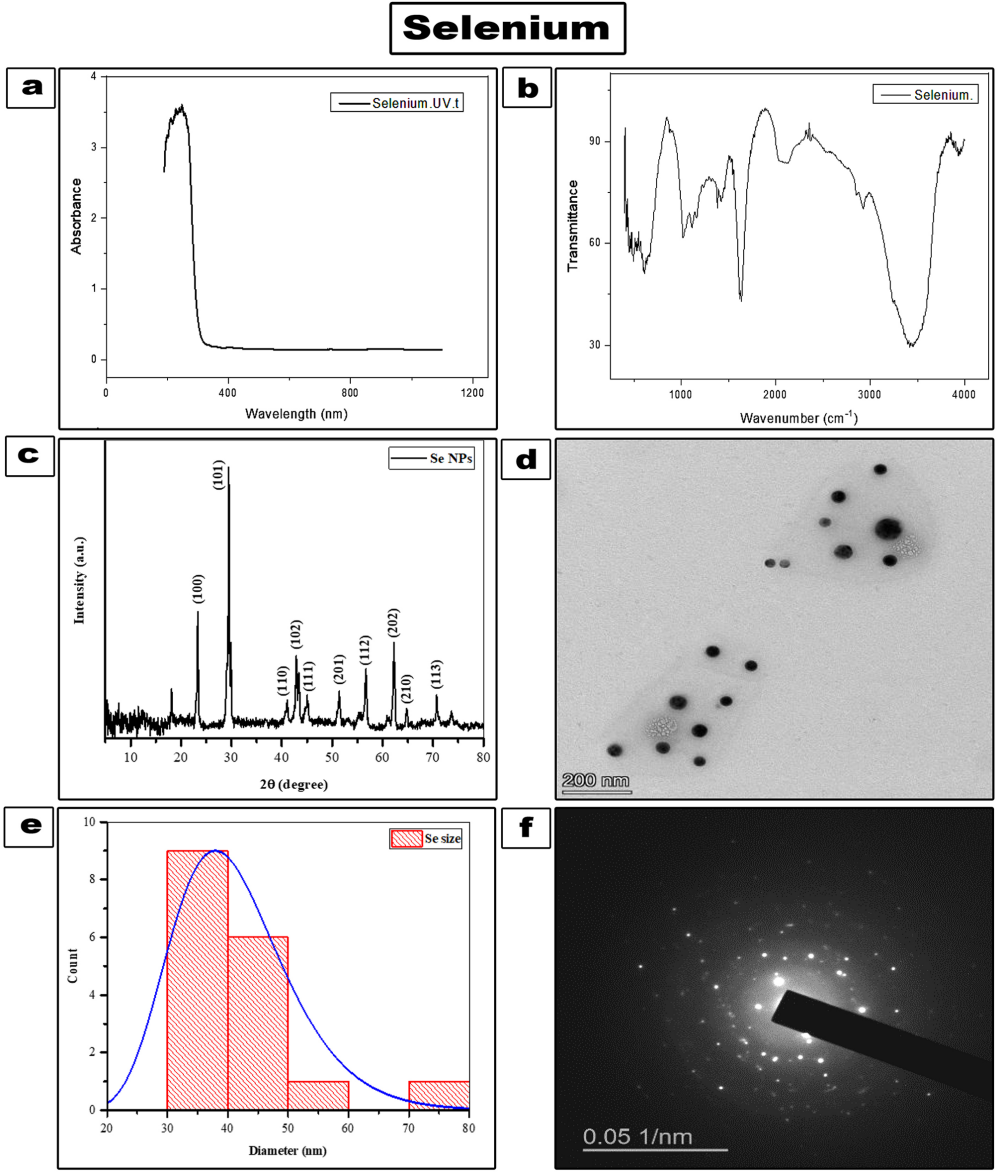
**(a)** UV–Vis absorption spectra of Se NPs **(b)** FTIR spectrum of crystalline SeNPs **(C)** XRD pattern of SeNPs **(d)** TEM image of SeNPs **(e)** Histogram distribution of SeNPs **(f)** Selected Area Electron Diffraction (SAED) pattern of SeNPs

UV-visible absorption spectra of SeNPs are shown in (Fig. 1a). The absorption peak for SeNps appears at 260 nm. This is related to interband and core electronic transitions [27]. FTIR spectroscopy has been employed to analyze the possible functional groups present in the produced nanoparticles. The FTIR spectrum was obtained from 400 to 4000 cm^− 1^ (Fig. 1b). The FTIR spectrum of SeNPs reveals several characteristic band peaks corresponding to different functional groups and interactions. The peak at 3450 cm⁻¹ is attributed to O-H stretching vibrations, while the peak at 2938 cm⁻¹ represents C-H stretching vibrations. The band at 1630 cm⁻¹ corresponds to C = O stretching vibrations. The peak observed at 1030 cm⁻¹ is associated with C-O stretching in alcohols. Finally, the band at 611 cm⁻¹ is significant as it indicates the coupling of SeNPs with the -OH group, demonstrating coordination linkages between selenium and ascorbic acid. These spectral features provide valuable insights into the molecular structure and interactions of the SeNPs [28–30]. X-ray diffraction was used to confirm the crystallinity of prepared SeNPs. (Fig. 1c) depicts the diffraction peaks of SeNPs over the diffraction angle 2θ of 5–80°. This verifies the creation of crystalline hexagonal SeNPs [31]. The crystalline size of the SeNPs has been calculated using Scherrer’s formula: [32]

$${\rm{D}}\, = \,{\rm{K \lambda /\beta }}\,{\rm{COS\theta }}$$

Where K is Scherer constant (0.9), λ is the X-ray wavelength, β is the width at half the XRD peak maximum, and θ is the Bragg angle. The crystallite size has been calculated and was found in the range of 40 nm for the Se-NPs.

TEM analysis was utilized to investigate the morphology and size of SeNPs (Fig. 1d). SeNPs are spherical particles with a homogeneous dispersion and a size of a few nanometers. Based on the histogram distribution (Fig. 1e), the particle size ranges from 20 to 80 nm. The Selected Area Electron Diffraction (SAED) pattern in (Fig. 1f) further indicates the high degree of crystallinity of Se NPs.

### Characterization of nano-filled resin composite after addition of Se NPs

#### Scanning electron microscopy with an energy-dispersive X-ray analytical system (SEM- EDX)

**Fig. 2:**
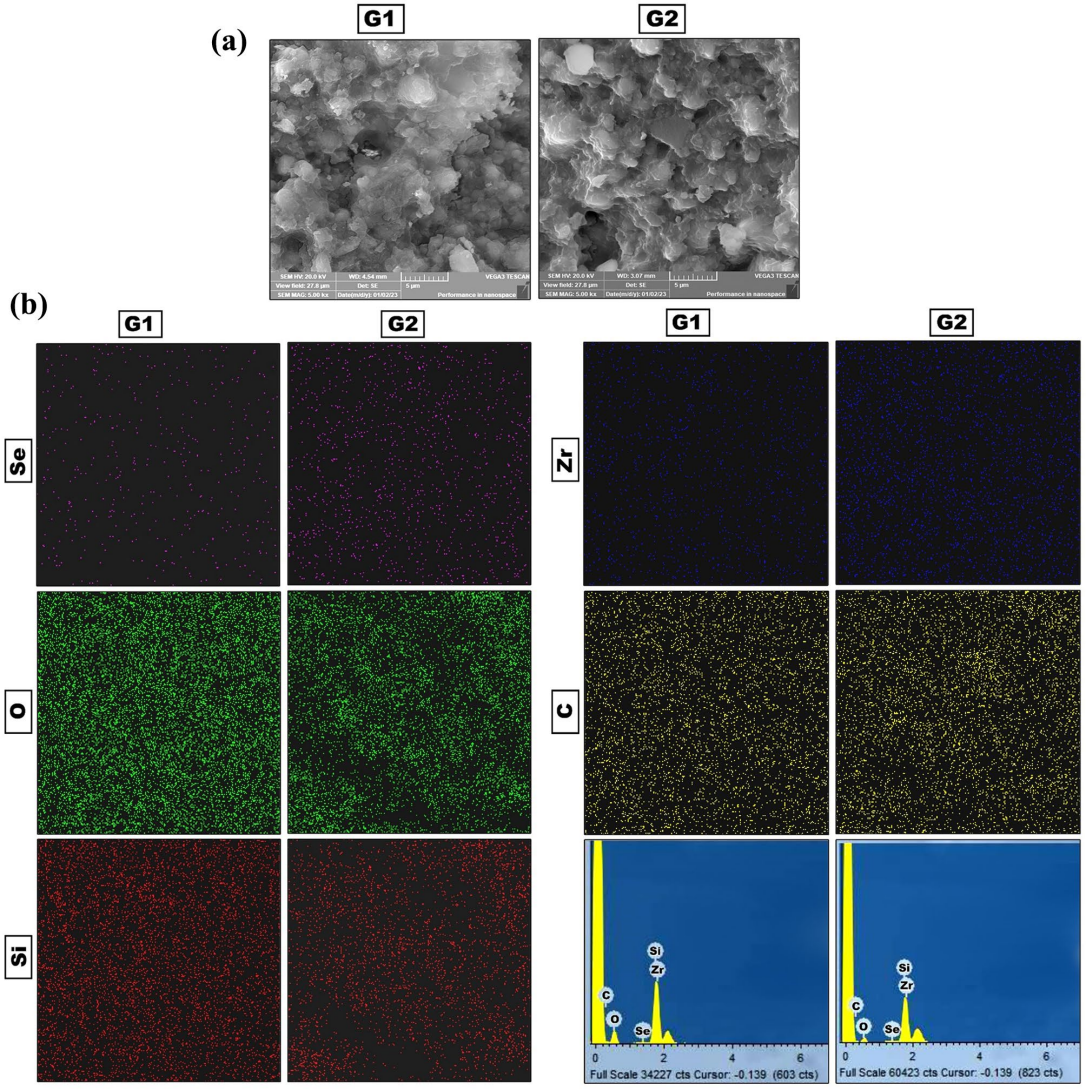
**(a)** Scanning electron microscopy (SEM) images of fractured surface of composite specimens modified by SeNPs (G1 and G2). **(b)** Energy-dispersive X-ray spectroscopy (EDX) mapping analysis of the fractured surface of composite specimens modified by SeNPs (G1 and G2)

Figure 2a showed SEM images of fractured surface of modified composite with SeNPs (G1 and G2). Figure 2b showed elemental mapping of Se, constituent elements of nanofilled composite material (O, Si, Zr and C) and EDX spectrum of the SeNPs modified composite (G1and G2). Elemental mapping showed uniform and wide elemental distribution of SeNPs in composite resin matrix in both SeNPs modified composite groups (G1 and G2). The EDX spectrum of the SeNPs modified composite showed the selenium nanoparticles displayed signals corresponding to elemental selenium confirming their presence as constituents without any impurities. Thus, EDX spectroscopy verified that the composition of nanoparticles aligned with expectations.

#### Antibacterial properties

Regarding the antibacterial activity of composite resin before and after addition of SeNPs, the means and standard deviations of inhibition zones against *E-coli* and *S. mutans* after 24 h of incubation are shown in Table 2. The direct contact test revealed that the incorporation of 0.005 and 0.01 wt% of SeNPs in composite resin provided a statistically significant antibacterial activity against *E. coli* and *S. mutans* (*p* < 0.05). Furthermore, increasing the amount of nanoparticles added to resin results in a statistically significant increase in inhibition zones.

**Table 2:** Means, standard deviations and results of Post Hoc test and one-way ANOVA of antibacterial activity of composite before and after addition of SeNPs against *E-coli* and *S. mutans*

GP/Conc % wt	Strain	Mean ± SD	*P* value
G0 G1 G2	*E-coli*	0.000 ± 0.00^a^ 11.612 ± 0.20^b^ 15.487 ± 0.36^c^	0.04^*^
G0 G1 G2	*S. mutans*	0.000 ± 0.00^a^ 15.718 ± 0.26^b^ 20.303 ± 0.28^c^	0.026*

Significance level ≤ 0.05

Means with the same superscript letter are not significantly different

#### Diametral tensile strength and compressive strength (MPa)

The mean values and standard deviations (MPa) for diametral tensile strength and compressive strength of composite without SeNPs (G0), composite modified by 0.005 SeNPs (G1) and composite modified by 0.01 SeNPs (G2) are presented in Table 3. There was no significant difference in diametral tensile strength and compressive strength after incorporation of nanoparticles into the composite resin (*p* > 0.05) compared to the unmodified group (G0).

#### Surface roughness (Ra)

**Fig. 3:**
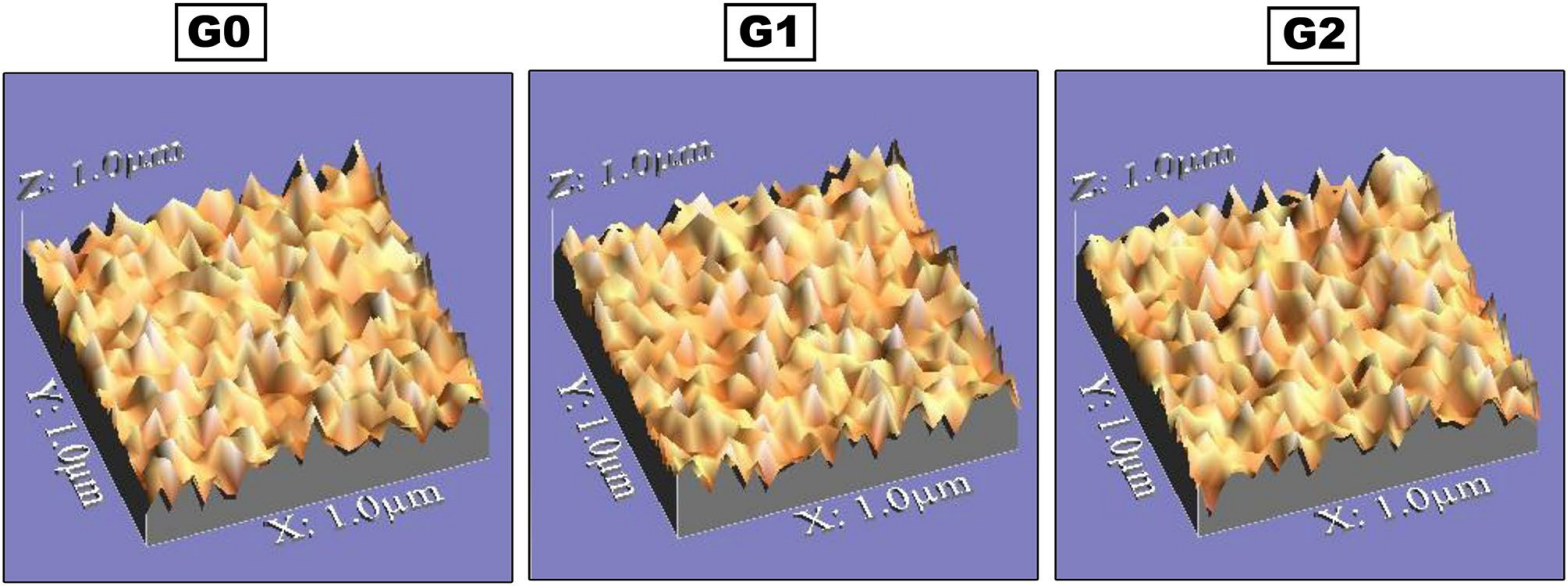
Surface roughness images of composite without SeNPs (G0), composite modified by 0.005 SeNPs (G1) and composite modified by 0.01 SeNPs (G2)

The mean values of the surface roughness (Ra) of the composite control group (G0) and composite resin Filtek™ Z350 XT modified by SeNPs (G1 and G2) are shown in Table 3. Representative surface roughness images of composite without SeNPs (G0), composite modified by 0.005 SeNPs (G1) and composite modified by 0.01 SeNPs (G2) are presented in Fig. 3. Statistical analysis showed no significant difference between modified and unmodified groups (*p* = 0.32) showing that the surface roughness of Filtek™ Z350 XT modified by nanoparticles was the same as the control unmodified group.

#### Degree of conversion (%)

The degree of conversion (DC%) of the composite control group (G0) and composite resin groups modified by SeNPs (G1 and G2) are displayed in Table 3. While the DC% of the composite resin modified by 0.005 wt% SeNPs is lower than that of unmodified composite, statistical analysis reveals no significant difference (*p* > 0.05) compared to unmodified composite.

**Table 3:** Means, standard deviations and results of Post Hoc test and one-way ANOVA of composite before and after addition of SeNPs regarding diametral and compressive strengths, surface roughness, degree of conversion and color difference

Tests	G0 (control) Mean±SD	G1 (composite modified with Se 0.005%) Mean ± SD	G2 (composite modified with Se 0.01%) Mean ± SD	*P*-value
Groups				
Diametral tensile strength	45.92 ± 2.06^a^	41.92 ± 0.95 ^a^	42.98 ± 1.016 ^a^	**0.29**
Compressive strength	192.2 ± 7.49 ^a^	181.5 ± 7.54 ^a^	182.3 ± 7.59 ^a^	**0.48**
Surface roughness	0.2897 ± 0.0023 ^a^	0.2890 ± 0.0029 ^a^	0.2884 ± 0.0036 ^a^	**0.32**
Degree of conversion	62.14 ± 1.10 ^a^	61.08 ± 1.79 ^a^	62.39 ± 1.32 ^a^	**0.38**
Color difference	--------------	15.32 ± 0.54 ^a^	15.45 ± 0.68 ^a^	**0.21**

Significance level ≤ 0.05

Means in the same row with same superscript letter are not significantly different

#### Color difference (ΔE)

The color difference values are listed in Table 3. The color difference mean value in comparison to (G0) was higher in composite modified by 0.01 SeNPs wt% (G2) than that recorded in 0.005 wt% SeNPsmodified composite (G1). However, statistical analysis showed no significant difference between modified tested groups (*p* = 0.21). There were obvious color changes in both composite groups modified with SeNPs (G1and G2) whatever the concentration of SeNPs (ΔE ≥ 3.3).

## Discussion

Dental plaque accumulates more favorably on the surface of restorative materials compared to enamel surface. Thus induced development of new dental restorative materials that contain antibacterial agents [33]. Antibacterial activity of Se-NPs has been reported against various microorganisms [34]. In previous studies, different nanoparticles were incorporated in composite resin improving their antibacterial properties without compromising the physical properties [19, 35, 36]. The choice of selenium nanoparticles and their concentration is driven by their effective antibacterial action, biocompatibility, and potential to enhance both the mechanical and physical properties of composite resins [37, 38]. 

Selenium has been prepared by several fabrication methods [39]. The simplest method includes chemical reduction of selenium salts for synthesis of Se-NPs [31]. As well, It can be synthesized biologically using microorganisms and plant extracts [40, 41]. It has been affirmed that the Se-NPs prepared from natural resources showed less toxicity than the Se nanoparticles prepared from chemical compounds [42, 43]. In this study, crystalline SeNPs green synthesis was performed successfully by using a chemical reduction method based on ascorbic acid (vitamin C) as a reducing agent. This makes the process economic, nontoxic and environment friendly producing spherical-shaped Se-NPs with a homogeneous dispersion without using any unfavorable chemical reducing or capping agents. SeNPs formation and stability were confirmed using UV-visible spectrophotometry. The absorption peak for SeNPs appears at 260 nm. FTIR shows the band at 611 cm^− 1^ corresponds to coupled SeNPs with the -OH group, demonstrating coordination linkages between Se and ascorbic acid. Thus, this indicates coating of SeNPs by ascorbic acid to avoid agglomeration. The FTIR spectrum study verifies the Se reduction [28]. XRD confirmed successful synthesis of hexagonal phase SeNPs [31].

TEM image of the prepared nanoparticles (Fig. 1d), indicates high surface area, allowing for greater contact area of nanoparticles with cell membrane of bacteria, producing bacterial cell damage [44]. SEM- EDX mapping of the fractured surface of composite resin showed homogenous and wide distribution SeNPs. EDX confirmed the elemental presence and purity of Se and O forming Se NPs. A small weight% of SeNPs 0.005wt% and 0.01 wt% were added to the nanofilled composite to avoid its aggregation or interference with curing light resulting in light reflection and scattering and lowering the degree of conversion [45]. 

Regarding the antibacterial activity of composite resin modified by Se-NPs, the findings of this study showed that SeNPs could inhibit *S. mutans and E-coli* even in small concentrations. Thus, Se NPs inclusion into the composite resin significantly inhibits *S. mutans* growth which is one of the main etiologic causes of caries formation. So, the first null hypothesis in this study was rejected. The antibacterial activity of selenium may be attributed to penetrating the cell wall, resulting in intracellular and extracellular morphological modifications in bacterial cells. After that, the formation of reactive oxygen species by selenium nanoparticles results in disruption of bilayer phospholipids where SeNPs inactivate intracellular proteins and denature sulfhydryl and the thiol groups present in membrane proteins. Hence, reactive oxygen induces cell death by either modifying the cycle of protein synthesis, intervening with the respiratory or food metabolism mechanism, or impeding DNA replication and causing oxidative stress [46]. 

To be an effective nanofiller, It should demonstrate improved antibacterial properties to inhibit biofilm formation without scarifying composite resin mechanical and physical properties [8]. composite resin should have good mechanical properties, especially in areas subjected to high masticatory stresses [47]. Thus, the incorporated antibacterial agents should not affect mechanical properties or preferably improve them. Regarding mechanical properties, our findings demonstrated no significant difference between unmodified composite and composite modified by SeNPs, demonstrating that the incorporation of small amounts, such as 0.005%–0.01 wt% of SeNPs, does not affect diametral tensile or compressive strength. The homogenous distribution of SeNPs within composite resin explains these findings. Other studies researched the addition of several nanoparticles in dental materials, including ZnO, and Se/ZnO NPs. They reported that nanoparticles incorporation doesn’t affect the mechanical strength [48, 49]. 

Surface smoothness is essential for successful restorations. Rough surface of the restorations favors plaque accumulation which in turn causes secondary caries with accompanied discoloration of the restoration and microleakage [50, 51]. Furthermore, a mean surface roughness of 0.3 μm can be easily identified by the tongue tip and thus makes patients feel discomfort. The range of mean surface roughness values obtained in this study is below 0.3 μm which is clinically acceptable [52]. The surface roughness revealed no significant difference after addition of SeNPs which confirms homogenous distribution of low concentrations of the Se-NPs within the polymer matrix.

The degree of conversion affects polymer molecular weight and therefore influences directly physical and mechanical properties. The degree of conversion of composite resin after addition of SeNPs remained unchanged as shown in Table 3. These findings are in alignment with previous study, which demonstrated no statistically significant differences in the degree of conversion following the addition of nanoparticles within the composite resin [53]. Different factors affect curing process such as the irradiation energy, the light wavelength, and depth of light penetration in resin composite. In addition, composite resin composition including filler particles amount and size impact degree of conversion. Furthermore, transparency of the resin matrix and fillers influenced light penetration [54]. SeNPs, being opaque, could increase opacity of composite resin and hinder the polymerization and reduce the degree of conversion. Nevertheless, SeNPs were added with low concentrations that allowed their dispersion into the composite resin and subsequently had no effect on degree of curing.

Different techniques have been utilized to accurately determine color alterations of composite restorations. Spectrophotometry is one of such techniques, that allows the analysis of numerous factors related to the color changes of composite resins. In this technique, reflected wavelength by a material is transformed to values expressed in ΔE. In this study, it was found that the modified composite with SeNPs exhibits a statistically significant increase in color difference values (ΔE) exceeding 3.3 and is visually distinguishable in both modified groups (G1 and G2). This could be explained by the difference in refractive indices between the selenium nanoparticles and organic matrix that impact the light refraction and reflection at the matrix-filler interface. The more the refractive indices difference relates to increased opacity of the resin composite regardless of nanoparticles type [55]. Thus, the second hypothesis was partially accepted where diametral strength, compressive strength, surface roughness, and degree of conversion were not changed after incorporation of either concentration of SeNPs. On the other hand, color difference (ΔE) is visually observed after the addition of both concentrations of SeNPs.

In the current study, there are some limitations to state. It is an in-vitro investigation that does not simulate a complex oral cavity environment. The release kinetics and leaching of Se ions were not studied. Furthermore, the thermal stability of nanoparticles needs to be explored in future studies. The evaluation of biofilm formation on modified composite needs to be considered in further studies. Additionally, further optimization of SeNPs filler can balance antibacterial effects and esthetic properties.

## Conclusion

The green synthesis of selenium nanoparticles and their incorporation as nanofillers into dental composite Filtek™ Z350XT led to significant enhancement of antimicrobial properties against *E. coli* and *S. mutans* bacteria. This was achieved without detrimental impacts on key mechanical/physical properties including diametral tensile strength, compressive strength, surface roughness, or degree of conversion. However, our study confirmed that there were significant differences in the color change of resin composite (ΔE).

## Data Availability

The datasets used and/or analyzed during the current study are available from the corresponding author on reasonable request.

## Abbreviations

SeNPs: Selenium nanoparticles
FT-IR: Fourier transform infrared spectroscopy
TEM: Transmission electron microscopy
PVA: Polyvinyl alcohol
XRD: X-ray diffraction
SEM: Scanning electron microscopy
EDX: Energy-dispersive X-ray mapping
BHI: Brain Heart Infusion
CIE: The International Commission on Illumination’s
BIS-GMA: Bisphenol A glycidyl methacrylate
UDMA: Urethane dimethacrylate
TEGDMA: Triethylene glycoldimethacrylate
BIS‑EMA: Bisphenol A ethoxylated dimethacrylate
SAED: Selected Area Electron Diffraction
DC: Degree of conversion
ΔE: Color difference
